# Functional recoding of *Chlamydomonas reinhardtii* thioredoxin type-h into photosynthetic type-f by switching selectivity determinants

**DOI:** 10.3389/fpls.2025.1554272

**Published:** 2025-03-06

**Authors:** Stéphane D. Lemaire, Gianluca Lombardi, Andrea Mancini, Alessandra Carbone, Julien Henri

**Affiliations:** ^1^ Sorbonne Université, CNRS, Laboratoire de Biologie Computationnelle et Quantitative LCQB, Paris, France; ^2^ Institut Universitaire de France, Paris, France

**Keywords:** photosynthesis, redox post-translational modification, thioredoxin, protein-protein interaction, functional determinants

## Abstract

Thioredoxins are ubiquitous disulfide reductases folded as an α/β domain of 100-120 amino acid residues. Functional redox site is composed of a pair of cysteines in a canonical WCGPC pentapeptide exposed at the surface of thioredoxins, that reduces disulfide bonds on target proteins. Several genetic isoforms of thioredoxins are phylogenetically classified into seven types, including type-h involved in general functions in the cytosol and type-f specifically associated to photosynthetic functions in chloroplasts. Specialization of thioredoxin function is correlated to its selectivity towards a type-dependent repertoire of protein targets. In this study, we combined biochemical and computational approaches to identify amino acid residues of photosynthetic type-f thioredoxin contributing to target the Calvin-Benson-Bassham cycle enzymes fructose-1,6-bisphosphatase and sedoheptulose-1,7-bisphosphatase. By introducing these residues into the scaffold of type-h thioredoxin, we generated a synthetic chimera of thioredoxin-h active towards photosynthetic fructose-1,6-bisphosphatase *in vitro*. Our combined computational and experimental approach provides a general pipeline for the design of molecular switches, enabling precise functional control.

## 1 Introduction

Thioredoxins (TRX) are small ubiquitous proteins functioning as dithiol-disulfide exchangers. Their highly conserved structure consists of a single globular domain of 3 layers, with a central mixed β-sheet of 4 strands in the order 4312, sandwiched between 2 pairs of α-helices (Qin et al., 1994). TRX tetrapeptide CGPC is exposed to solvent where, in the reduced state, the first cysteine of the motif is available for reduction of a disulfide on a target protein (Eklund et al., 1992). Crystal structure of a TRX-target complex points to the importance of recognition features around protein disulfides, which in the case of HvTRXh2 and α-amylase/subtilisin inhibitor (BASI) relies on van der Waals contacts and backbone-backbone hydrogen bonds (Maeda et al., 2006). TRX are encoded in every organism, and their phylogenetic history enabled the characterization of TRX redox function in molecularly resurrected extinct versions (Perez-Jimenez et al., 2011). Strikingly, crystal structure determinations of both extant and inferred extinct versions confirm the strict conservation of TRX fold over 4 billion years of molecular evolution (Ingles-Prieto et al., 2013). Meanwhile, the evolutionary requirements of TRX mirrored the emergence of new protein targets, *e.g.* from the universal ribonucleotide reductase (Laurent et al., 1964) to more specific biological functions.

*Viridiplantae* notably diversified their TRX encoding genes into an apparently complex multiplicity (Lemaire and Miginiac-Maslow, 2004). In the green model microalga *Chlamydomonas reinhardtii* (from now on Chlamydomonas), 5 types of thioredoxins co-exist in the choroplast: TRXf1-TRXf2, TRXm, TRXx, TRXy, TRXz (Lemaire et al., 2007). Structural comparison of nuclear magnetic resonance or crystal structures and computational models confirmed the strong structural similarities among Chlamydomonas TRX and revealed a diversity of local repartition in surface electrostatics (Lancelin et al., 2000) (Lemaire et al., 2018) (Le Moigne et al., 2021) consistent with the hypothesis of a selectivity code for target recognition primarily based on electrostatic complementarity (Bunik et al., 1999). Indeed, several enzymes involved in chloroplast photosynthetic metabolism are activated by disulfide reduction (Jacquot et al., 1984) (Decottignies et al., 1988) and notably Calvin-Benson-Bassham cycle (CBBC) enzymes for carbon fixation (Schürmann and Buchanan, 1975) (Schürmann and Wolosiuk, 1978), hence conditioning energy-consuming anabolic carbon fixation to the reducing activity of illuminated photosystems (Droux et al., 1987) (Buchanan, 2016). Among these CBBC targets, fructose-1,6-bisphosphatase (FBPase) and sedoheptulose-1,7-bisphosphatase (SBPase) were the focus of several *in vitro* studies to demonstrate TRXf selectivity for both enzymes FBPase and SBPase (Yoshida et al., 2015). Electrostatic-based selectivity of TRXf for FBPase is strenghtened by the proteomic enrichment of FBPase on TRXf affinity chromatography (Balmer et al., 2004). Besides, electropositive surface is partially shared between TRXf2 and TRXm, *eg.* for the recognition by both types of the chloroplast ATP synthase (Sekiguchi et al., 2020).

In this study, we developed a computational strategy to identify functional determinants of TRXf specificity for FBPase; consistently with previous work TRXf2 functional determinants of specificity are electrostatic groups from surface-exposed residues. We demonstrated their effect on target recognition in a synthetically re-addressed TRXh. We used cytosolic TRXh1 (Marchand et al., 2019) from *Chlamydomonas reinhardtii* for the target substitution of 5 lysines at previously neutral to electronegative character. Induced selectivity for FBPase was then probed with pure recombinant protein, monitoring the redox reactivation of FBPase. We observed that the chimeric TRXh1 with selectivity motifs from TRXf2, called TRXχ1, indeed gained the selectivity towards FBPase *in vitro*.

We demonstrated the successful engineering of a synthetically re-addressed TRXh, showcasing our ability to reprogram target recognition through engineered specificity *in vitro*. Building from this study-case, we propose a combined computational and experimental pipeline for the design of molecular switches, enabling precise functional control. Our results highlight the major contribution played by TRX surface electrostatics for the selective protein-protein recognition towards a CBBC target.

## 2 Materials and methods

### 2.1 Plasmids and cloning material.

Protein coding sequences were cloned into pET bacterial expression plasmids in amino-terminal fusion with poly-histidine tag enabling Nickel-affinity chromatography. Cloning, expression and purification of CrTRXf2 and CrTRXh1 were previously published and repeated without changes (Lemaire et al., 2018) (Marchand et al., 2019). Sequence encoding TRXχ1 was synthesized *de novo* and cloned in pET28 in between NdeI and BamHI. Inserted nucleotide sequence is: 5’- CATATGGGCGGTTCTGTTATTGTGATCGACTCCAAGGCGGCTTGGGACGCTCAGCTGGCCAAGGGCAAGGAGGAGCACAAGCCGATTGTTGTCGACTTCACTGCTACGTGGTGCGGTCCTTGCAAGATGATTGCGCCGCTGTTTGAGAAGCTGAGCAACGACTATGCTGGCAAGGTCATCTTCCTGAAGGTCGATGTTGACGCCGTCAAGAAGGTTGCCGAGGCTGCCGGCATCAAGGCCATGCCCACCTTCCATGTGTACAAGGATGGCGTGAAGGCGGACGACCTAGTGGGCGCCAAGCAGGACAAGCTCAAGGCCCTGGTCGCCAAGCACGCCGCCGCGTAAGGATCC-3’. It codes for the full-length version of CrTRXh1 (113 amino acid residues, 12 kDa) fused to a cleavable amino-terminal hexahistidine tag. Five substitutions were made at T49K, A69K, A70K, T78K, S99K (numbering according to Protein Data Bank entry 6I19) in order to insert positively charged groups.

### 2.2 Recombinant protein preparations

Plasmids coding for CrTRXf2, CrTRXh1, TRXχ1, and CrFBPase were transformed into competent *Escherichia coli* BL21(DE3) for inducible overexpression. At late exponential phase in 1-L antibiotic-selective LB culture, expression was started with 0.2 mM isopropyl-β-D-thiogalactopyranoside (IPTG) and incubation for 3h at 30°C. Cells were collected by centrifugation and the pellet was resuspended in 20 mM Tris-Cl pH=7.9, 100 mM NaCl (buffer A) and lysed by 3 cycles of 1 min sonication in pulses of 1 s followed by 1 s pauses, yielding total lysates (T). Soluble fraction (S) was obtained by centrifugation at 21,300 × *g* and loaded onto NiNTA resin (Sigma-Aldrich). After washing the resin with buffer A and buffer A supplemented with 20 mM imidazole, the purified proteins were eluted by buffer A supplemented with 200 mM imidazole (E). Purified proteins were concentrated by ultrafiltration, tested for activity in the following week, and stored at -20°C until further use. As previously reported from us among others, recombinant purified TRX are stable over long experimental time *eg.* crystallization (Lemaire et al., 2018) (Marchand et al., 2019) (Le Moigne et al., 2021).

### 2.3 Enzymatic assays

**Figure 1:**
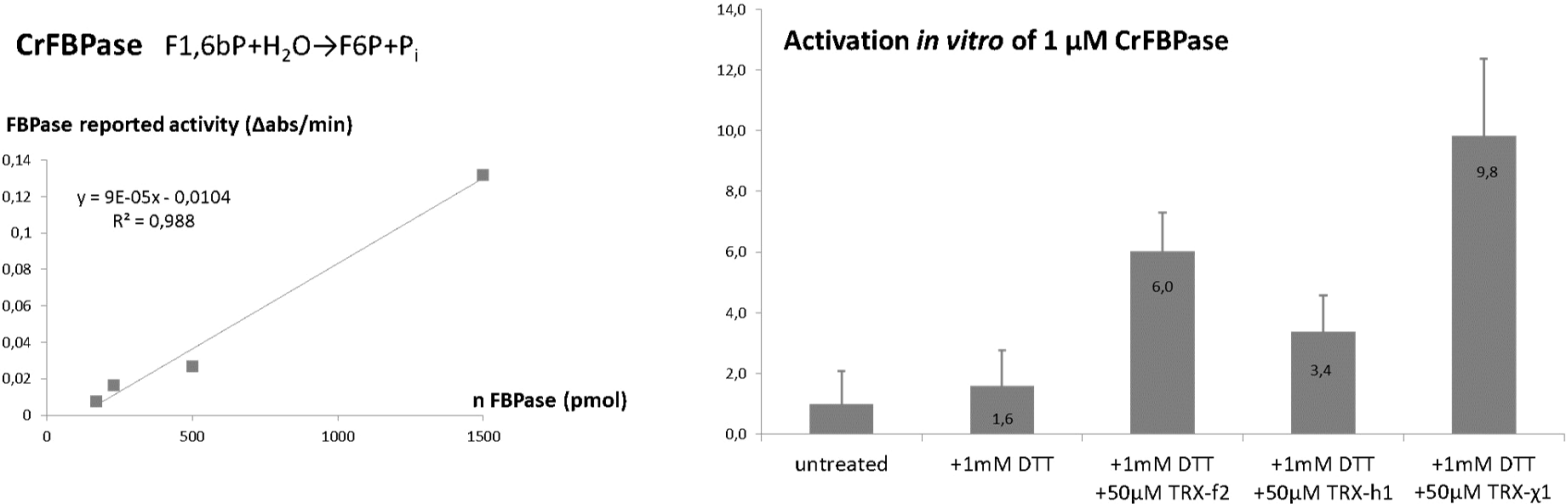
TRXχ1 functionally mimics f-type thioredoxin. (Left) Spectrophotometric reporter assay for FBPase was tested for linearity. (Right) FBPase reported activity under redox treatments: untreated, 1 mM dithiothreitol (DTT), 1 mM DTT and 50 µmol/L of CrTRXf2/CrTRXh1/TRXχ1.

The activity of FBPase was determined in the direction of FBP hydrolysis in a reporter coupled system via spectrophotometry on Uvikon-XS (Secomam, 500 µL reaction mix) at a room temperature controlled by 20°C air-conditioning, or Clariostar 96-well plate reader thermostated at 20°C (BMG Labtech, 100 µL reaction mix). 1 µL of pure recombinant CrFBPase concentrated at 254 µM was pre-incubated at 20°C for 5 min diluted in a reducing mix containing 1 mM dithiothreitol (DTT) and 50 μM TRXf2/TRXh1/TRXχ1 and buffered with 20 mM Tris-Cl pH=7.9, in a reaction volume of 10 µL. 1 µL of FBPase mix post-incubation was then assayed at 20°C for 120 min in a reaction mix containing 0.2 mM NADP^+^, 30 mM Tris pH 7.9, 10 mM MgCl_2_, 0.6 mM FBP, 0.1 unit of glucose 6-phosphate dehydrogenase, and 0.1 unit of phosphoglucose isomerase. Reduction of NADP^+^ was followed at 340 nm at 20°C and the slopes representing initial velocities (*v*_i_) were extracted. Each assay was replicated 4 times (n=4). Results are reported in **Figure 1** as a histogram of average *v*_i_ ± standard deviation for each condition. Statistical significance was probed by analysis of variance.

### 2.4 Modeling of protein structures

Protein sequences were used for computational prediction of TRXχ1 and for its complex with CrFBPase in https://alphafoldserver.com/ accessed on June 2024 (Abramson et al., 2024). Full-length proteins were given, with one Magnesium ion expected to map into reduced CrFBPase active site (Chiadmi et al., 1999). Models were checked for accuracy with pTM, iPTM, and pLDDT. Overall, protein folds for TRXχ1 and CrFBPase cores were robustly predicted (plDDT>0.9) although local segments of uncertainty appear (0.7>plDDT>0.5), notably at the TRX-CrFBPase interface in the predicted complex.

### 2.5 Computational search for selectivity determinants

To identify selectivity determinants, we employed two computational methods: ProfileView (Vicedomini et al., 2022) and MuLAN (Lombardi and Carbone, 2024), which revealed several insights into TRX and its functional properties.

ProfileView is a computational method designed to classify protein sequences into functional classes and subclasses, effectively handling datasets containing up to a few thousand sequences. This unsupervised method highlights the necessity of annotating multiple sequences simultaneously rather than individually, facilitating the discovery of novel functional classes and alternative specific functions. By extracting critical information, ProfileView identifies single essential residues that are key to the function of a specific subclass. These residues serve as functional signatures for proteins; in enzymes, they are typically linked to catalytic activity and substrate selectivity.

MuLAN (Mutation-driven Light Attention Networks) is a deep learning method that predicts mutational effects on protein-protein interactions and identifies interaction surfaces. It reconstructs the mutational landscape of a protein in relation to a given interaction partner. MuLAN mutational scores are averaged across all possible mutations at each position to derive a significance score for individual residues.

### 2.6 TRX analysis with ProfileView

Using ProfileView, we analyzed over 10,000 TRX sequences and identified eight distinct functional classes. This classification was achieved by defining a multidimensional functional space (Steinegger and Söding, 2017), where hierarchical clustering revealed eight groups of TRX sequences. One of these classes, termed TRX-hfo, comprises 301 sequences and includes all known f-type sequences alongside certain h-type and o-type TRX sequences. Within the TRX-hfo group, the f-type sequences form a more refined subset of 34 sequences, designated TRXf. Aligning the TRXf sequences (**Supplementary Figure S1**) allowed us to investigate conserved positions, where we observed a significant conservation of lysines within this subset.

### 2.7 TRX analysis with MuLAN

MuLAN takes single interacting sequences as input, and we used it to compute the mutational landscape of TRXf2 in association with CrFBPase. In this analysis, scores were normalized to the range [0,1] using rank sorting, and predictions for the interaction surface were also obtained.

The MuLAN scores were mapped onto the TRX structure by averaging the predicted impact of all possible mutations at each position. This average serves as a measure of the *significance* of each position, highlighting residues that are critical for TRX activity and binding.

## 3 Results

### 3.1 Computational search for selectivity determinants

**Figure 2:**
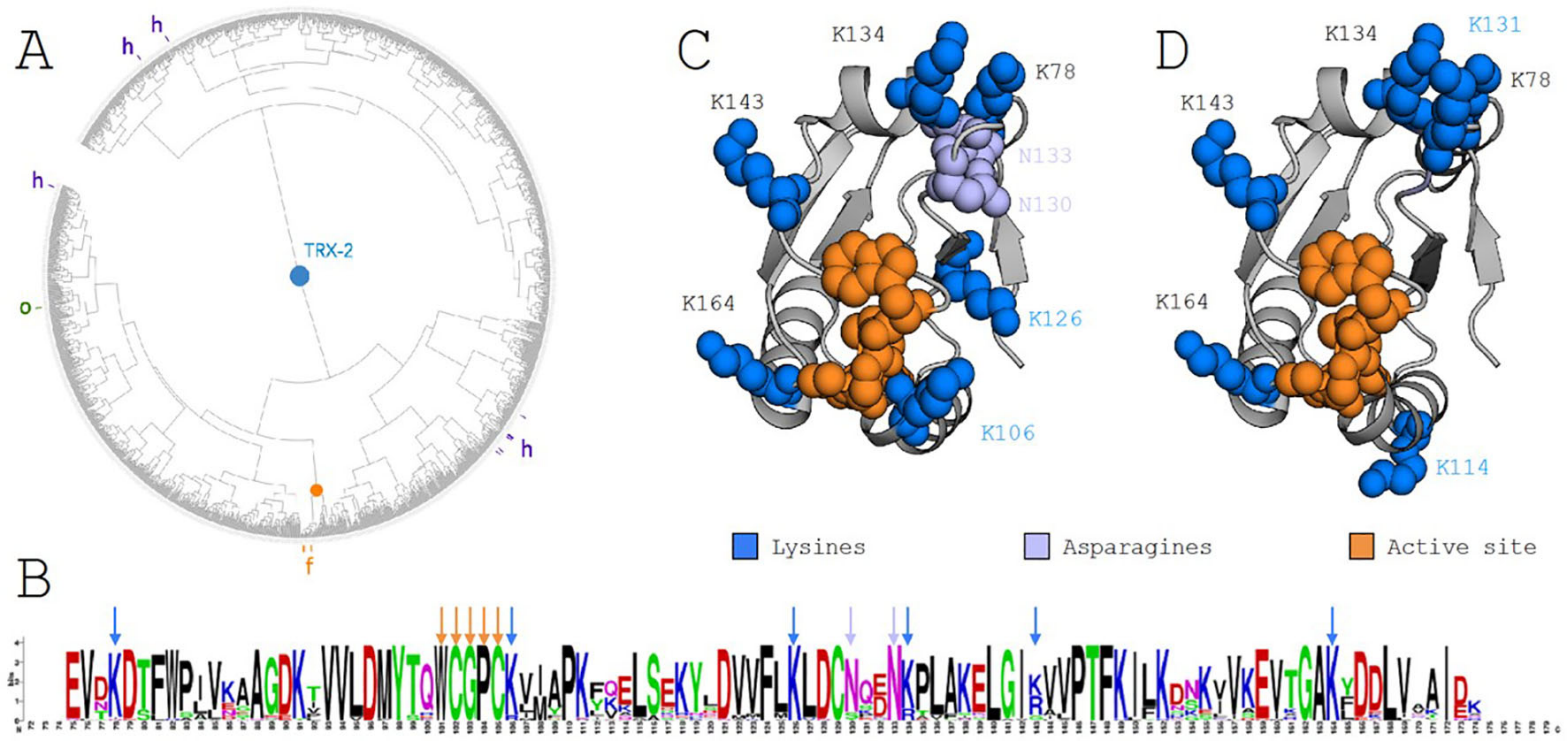
ProfileView analysis of the thioredoxin family and the thioredoxin type-f subfamily. **(A)** ProfileView tree of the TRX subfamily, comprising 301 sequences. The orange root marks the position of known TRX type f (TRXf2) sequences from *C*. *reinhardtii*. The tree was visualized using iTOL (https://itol.embl.de). **(B)** Motif derived from the alignment of sequences within the subtree highlighted by the orange root in **(A)**, generated using WebLogo (https://weblogo.berkeley.edu/logo.cgi). Orange arrows denote the conserved binding motif WCGPC. Blue arrows highlight conserved lysines (K) present in the *C*. *reinhardtii* TRXf2 sequence, while purple arrows indicate two conserved asparagines (N). **(C)** The TRXf2 structure (PDB 6I1C) showing residues involved in the functional determinant of the *C*. *reinhardtii* sequence. The extended disulfide bond motif (WCGPC) is highlighted in orange. A positively charged crown surrounding the active site, composed of six lysines (blue) and two asparagines (purple), is shown, with all other residues in gray. **(D)** Residues mutated in the switch experiment are highlighted in blue. The extended WCGPC motif (orange) indicates the location of the mutated residues forming the positive electrostatic crown. Note that K114 and K131 were mutated in place of K106 and K126, as shown in **(C)** (see text for details).

Designing enzymatic switches poses a significant challenge due to the difficulty in identifying the residue signatures responsible for enzymatic function and substrate recruitment. To address this, we applied ProfileView, a computational method that determines the functional signature of the TRXf2 sequence by analyzing a diverse set of TRX sequences found in nature. ProfileView leverages the evolutionary trajectories of natural sequences to classify TRX proteins based on their functional characteristics. When applied to over 10,000 TRX sequences, ProfileView identified eight major functional classes, each predicted to carry out distinct functions. One of these classes contains 301 sequences, encompassing all known type-f sequences along with several characterized type-o and type-h sequences. **Figure 2A** presents a functional tree illustrating the distribution of the 301 sequences within the ProfileView sequence space, which, it is important to note, does not represent a phylogenetic space. As shown in the tree, all annotated type-f sequences cluster tightly together, forming a subset of 34 type-f-like sequences. The identification of these type-f-like sequences was pivotal for our experimental design, as it enabled us to detect a functional signature consisting of six lysines (namely K78, K106, K126, K134, K143, K164) in the sequence CrTRXf2 which were conserved for the 34 type-f-like sequences (see logo in **Figure 2B**). These residues form a crown of electropositive charges surrounding the conserved active site of the TRX family (**Figure 2C**).

**Figure 3:**
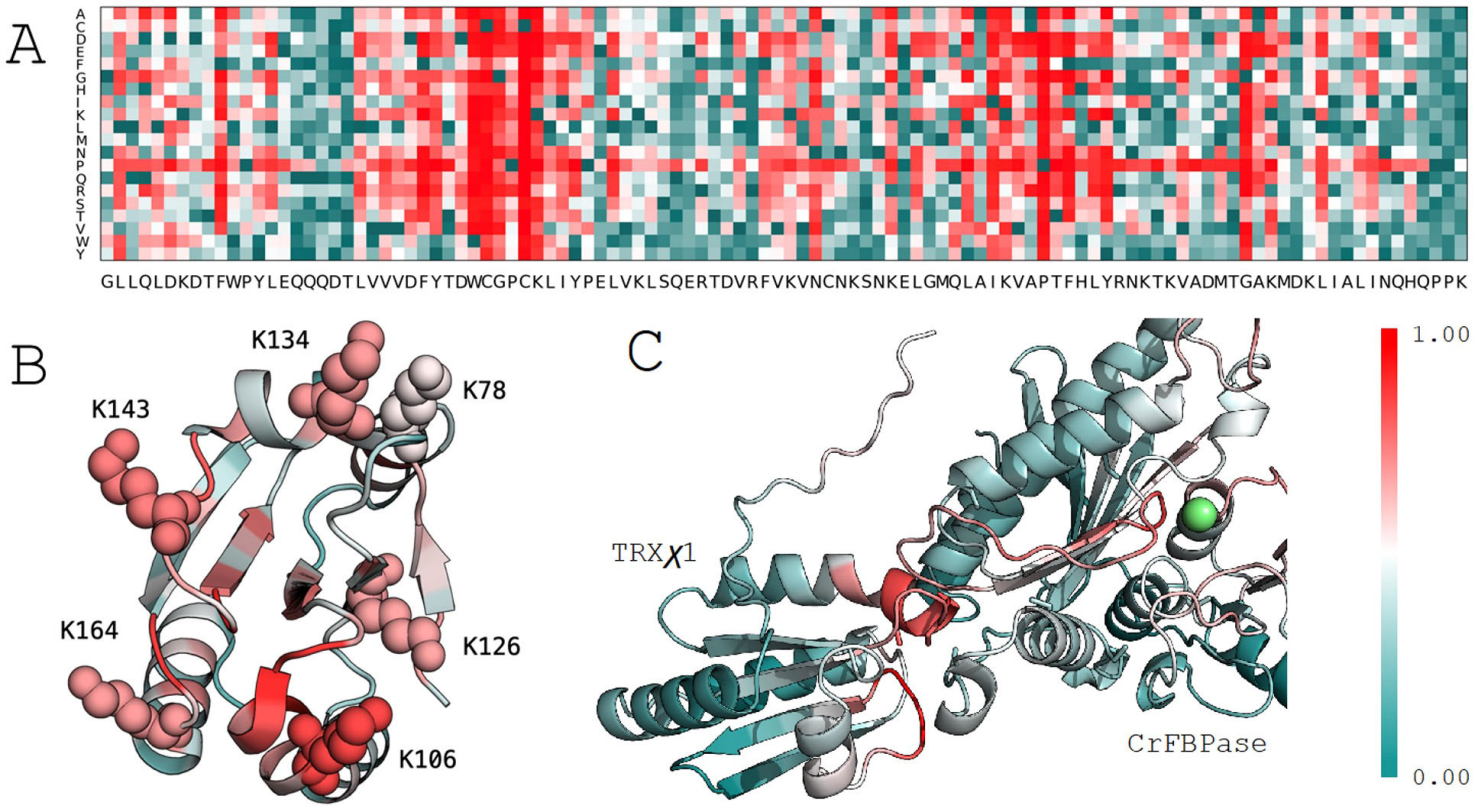
MuLAN analysis of thioredoxin type f (CrTRXf2) in *C*. *reinhardtii*. **(A)** MuLAN reconstruction of the mutational landscape for CrTRXf2. Each residue in the sequence is mutated, and the predicted effect is scored and visualized with a color gradient (red: high effect, green: no effect). **(B)** Highlighting of the six lysines identified by ProfileView, with their corresponding MuLAN scores. **(C)** MuLAN attention scores mapped onto the AlphaFold structural model of the TRX-FBPase complex. The magnesium ion is depicted in green.

To prioritize which of these lysines to mutate in the design of our functional switch, we employed MuLAN, a deep learning architecture developed to estimate changes in binding affinity within a protein complex. MuLAN merely uses single protein sequences and encodes them into embeddings using the Ankh protein language model (Elnaggar et al., 2023) to reconstruct the mutational landscapes of interacting proteins. Specifically, MuLAN was applied to TRX in association with CrFBPase, and the complete mutational landscape for CrTRXf2 is illustrated in **Figure 3A**, where the most sensitive mutations are highlighted by the highest scores (in red) assigned to residues in the core of the protein, fundamental for the TRX fold stability, in the WCGPC active site on the surface, and also in a small PTF motif, at positions 146-148, that is structurally close to the active site. The average per-position scores of the reconstructed mutational landscape were mapped onto the TRX structure (PDB 6I1C) (**Figure 3B**). This mapping reveals that the six lysine residues identified in the ProfileView analysis are also predicted to be highly significant by MuLAN (**Figure 3B**) and highly sensitive to mutation (*ie*. determining function). In particular, lysines were assigned higher scores with respect to surrounding residues. As an independent validation of the MuLAN prediction, we used AlphaFold3 to predict the structure of a binary complex between TRX and FBPase (**Figure 3C**), as detailed thereafter.

### 3.2 Design: thioredoxin synthetic selectivity determinants

Computationnally revealed thioredoxin-f selectivity positions for the redox activation of CBBC enzymes were mapped onto the crystal structure of two TRX from *Chlamydomonas reinhardtii* (Cr), a model green microalga. The representative structure of the f-type is that of chloroplastic CrTRXf2 (PDB 6I1C) while we took cytosolic CrTRXh1 structure (PDB 6I19) as representative of a non-photosynthetic type. Indeed, TRXh1 is representative of cytosolic isoforms found in eukaryotes and not directly involved in photosynthesis [for review: (Zaffagnini et al., 2019)], while TRXf2 is involved in the activation of enzymes in the CBBC [for review: (Michelet et al., 2013)].

**Figure 4:**
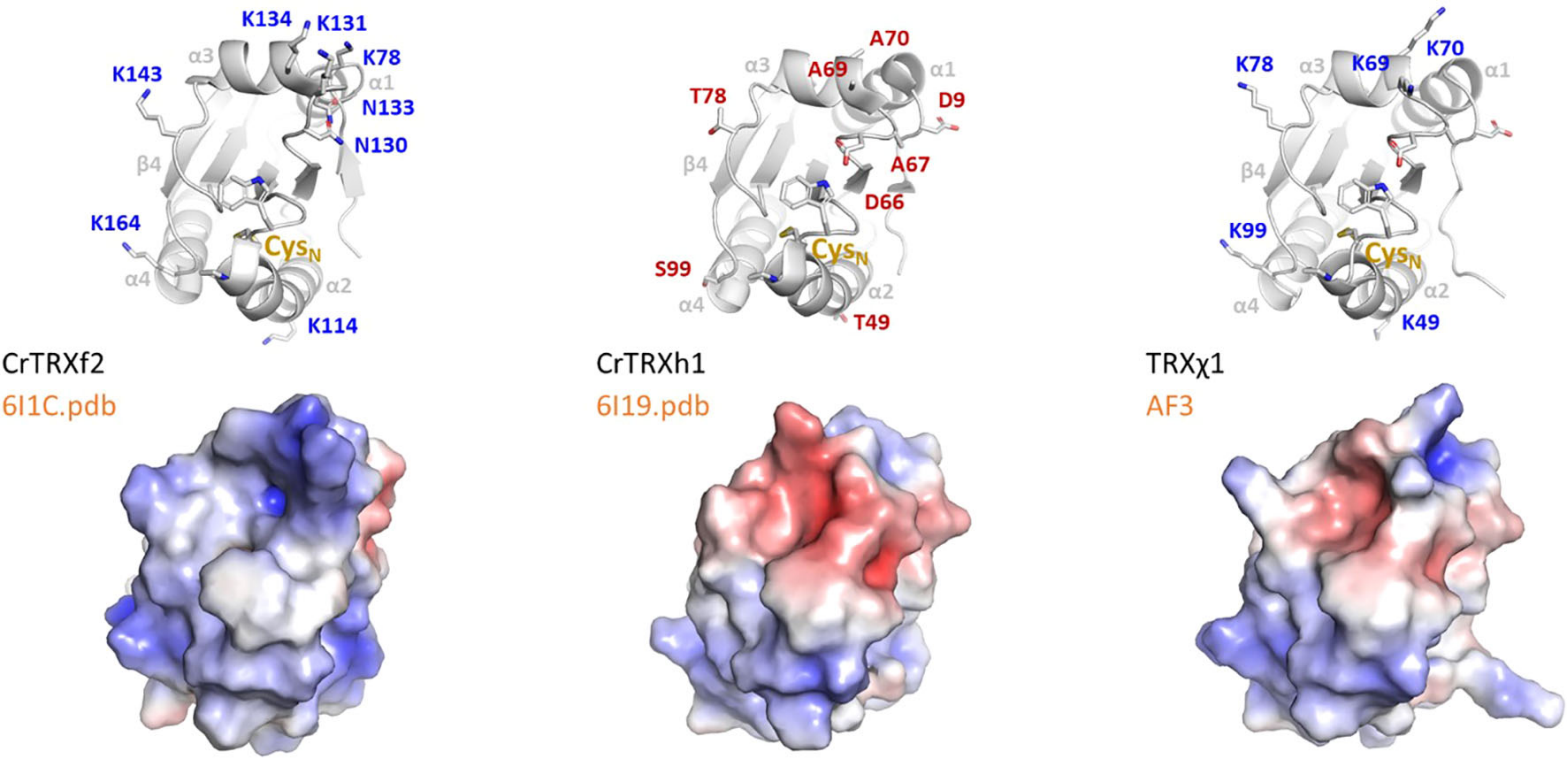
Electropositive groups form a conserved f-type around redox site. (Left) CrTRXf2 from crystal structure 6I1C, (center) CrTRXh1 crystal structure 6I19, (right) TRXχ1 model structure from AlphaFold3. (Top) Main chains are represented in cartoon with WCGPC motif and electro-selective, solvent exposed residues sidechains in sticks; (bottom) electrostatic surface potential from PyMOL APBS is colored from blue-electropositive to red-electronegative.

Sequence from Uniprot entries A0A2K3DSC9 (CrTRXf2) and P80028 (CrTRXh1) align with 40% identity, with 34/84 aligned residues and 2 gaps of the 180 and 133 total length of the queries. Meanwhile, CrTRXf2 and CrTRXh1 crystal structures superpose with an RMSD=0.953 Å, showing that structural similarity somewhat dominates sequence divergence. Redox motif CGPC is identically positioned in aligned structures, both TRX being in the oxidized state. The main, still moderate, differences map to (1) helix α1 that is longer by 2 turns in TRXh1 compared to TRXf2, (2) helices α3 and α4 that are slightly more compacted towards the hydrophobic core in TRXf2, and (3) loops immediately upstream of helices α1 and α3 that flap their main chains away from each other in TRXf2 and TRXh1 by 3 Å and 6 Å, respectively. In spite of these local divergences, TRXf2 and TRXh1 appear mostly identical from the standpoint of fold and main chain conformations (**Figure 4**, top). Rather, electrostatic potential at solvent-exposed surface maps a distinct distribution of charges between CrTRXf2 and CrTRXh1. Overall, CrTRXf2 appears electropositive around its redox reactive site while CrTRXh1 displays a mixed, rather electronegative character (**Figure 4**, bottom). Surface topology also appears partially remodeled, notably at the β-carboxy group of D66 (aspartate) that bridges a gap between helix α3 and the redox reactive site in CrTRXh1.

From the alignment of structures, we visually pinpointed which residues in CrTRXf2 would contribute an electropositive potential where equivalent residues of CrTRXh1 would confer a neutral or electronegative potential. We listed K78 (lysine), K114, N130 (asparagine), K131, N133, K134, K143, K164, as electropositive contributors to TRXf2 surface, and D9, T49 (threonine), D66, A67 (alanine), A69, A70, T78, S99, as neutral or electronegative contributors to TRXh1 in near 3-dimensional positions.

### 3.3 Build: f-type thioredoxin on an h-type scaffold

**Figure 5:**
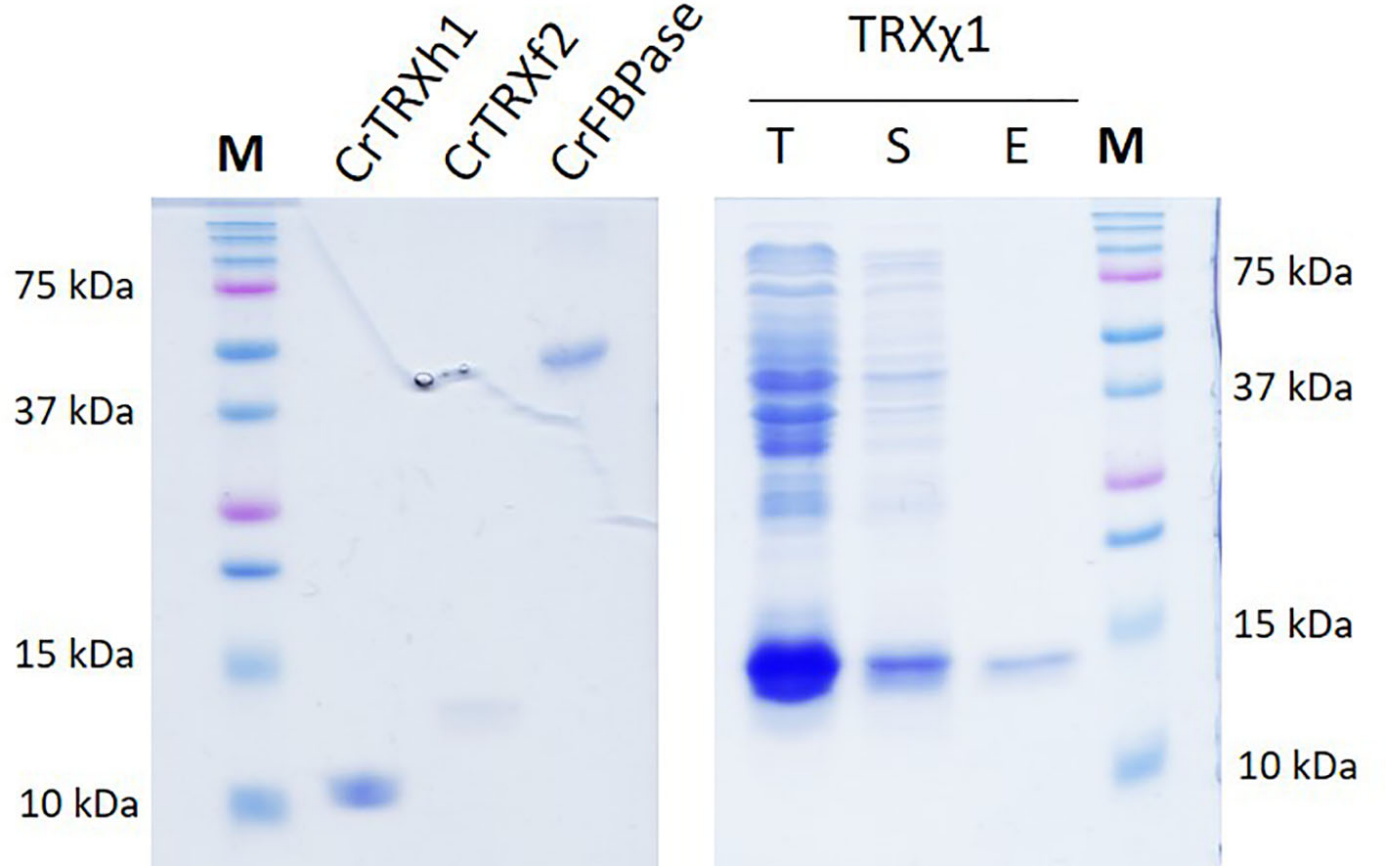
TRXχ1 recombinant protein purification. CrTRXh1 is mutated into CrTRXχ1 with 5 point substitutions: T49K, A69K, A70K, T78K, S99K. Denaturing gel electrophoresis (SDS-PAGE) of purified CrTRXh1, CrTRXf2, CrFBPase, and purification steps of TRXχ1 were loaded for separation and stained with Coomassie blue. T, total bacterial lysate; S, soluble fraction of lysate; E, Nickel-affinity chromatography eluate.

In order to test the deterministic character of electrostatics in conferring TRX-selectivity, we designed a modified version of CrTRXh1 that would be grafted with electropositive determinants from TRXf2. We chose positions T49, A69, A70, T78, S99 for grouped mutagenesis, substituting lysines in these 5 sites. Resulting modified TRXh1-K49,K69,K70,K78,K99 was named chimera 1 (TRXχ1) to account for its dual identity of a TRXh1 structural scaffold including native redox reactive site, and 5 electropositive group added around the redox site. Nucleotide sequence encoding TRXχ1 was synthesized *de novo* and cloned by insertion-ligation in a routine bacterial expression plasmid. Purified protein behaved as any TRX (**Figure 5**) and could be used as a probe for the redox activation of a CBBC target *in vitro*.

### 3.4 Test: induced recognition on f-type canonical target fructose-1,6-bisphosphatase

Purified CrFBPase was used a reporter of TRX activity. Recombinant protein was added to a standard spectrophotometric assay to quantitatively measure the level of fructose-1,6-bisphosphate hydrolysis over time. FBPase alone has an intrinsically low activity, expected when the inhibitory disulfide bridge constrains the active site into a low-activity form (Chiadmi et al., 1999) (Ke et al., 1990). Pre-incubation of CrFBPase with a low concentration of reducing agent dithiothreitol (DTT, 1 mM: *v*_i_^FBPase,DTT^ = 28 ± 20 ΔAbs/min) slightly activated the enzyme, but the addition of CrTRXf2 in the pre-incubation condition increased FBPase activity by 6-fold (*v*_i_^FBPase,DTT-TRXf2^ = 106 ± 22 ΔAbs/min). Cytosolic, non-FBPase selective TRXh1 only activated FBPase by 3.4-fold (*v*_i_^FBPase,DTT-TRXh1^ = 59 ± 21 ΔAbs/min) while its TRXχ1 counterpart replicated and exceeded TRXf2 activation, at a 9.8-fold increase of enzyme activity (*v*_i_^FBPase,DTT-TRXχ1^ = 172 ± 45 ΔAbs/min) (**Figure 1**). Analysis of variance between TRXh1- and TRXχ1-treated FBPase yield a p-value = 3,76.10^-3^ that we interpret as a significant difference. We conclude that *in vitro*, our modified version of TRXh1, substituted with 5 electropositive groups at f-type positions, gained the ability to interact productively with FBPase for its activation by reduction.

The transitory TRX-FBPase complex required for reduction of the regulatory disulfide bridge could be modeled by Alphafold3. In the predicted complex, the redox reactive center of TRXχ1 is placed in close proximity to CrFBPase regulatory cysteines, mapping a plausible interaction state during FBPase reduction process (**Figure 3C**). MuLAN attention scores have been plotted on the generated AlphaFold structural model, where we observe that the binding site of the TRX protein is correctly highlighted in red, as sensitive, and that a cysteine-rich motif lies in the interaction site as expected. As for the CrFBPase, one sees red residues lying at the interaction site and near catalytic site magnesium atom (in green). This photosynthetic-insertion loop is the allosteric determinant for FBPase activation in response to reduction of the inhibitory disulfide bridge (Chiadmi et al., 1999) (Villeret et al., 1995). It hence appears that ProfileView-MuLAN applied to the redox couple TRXf/FBPase enabled both the detection of protein-protein selectivity determinants, and the structurally dynamic relay loop formed by residues E166-T175 (numbering as in Uniprot entry A8IKQ0|CrFBPase, E165 contributes to Mg binding) that allosterically activates the enzyme.

## 4 Discussion

From a methodological perspective, this study highlights the utility of ProfileView, a tool for functional classification of protein sequences, in identifying crucial functional signatures for protein activity, here in the selective recognition of a cognate protein surface. By mapping residues in a target sequence to those conserved within functionally similar sequences identified by ProfileView, we pinpointed key lysines essential for the function of CrTRXf2. This demonstrates not only the effectiveness of ProfileView in elucidating protein-specific features but also its general potential for studying proteins with other diverse and specialized functions. The versatility of this method opens exciting possibilities for the rational design of proteins, where known functions can be fine-tuned or even re-engineered for entirely new applications. While CrTRXf2, a well-characterized type-f sequence, served as our model, the broader thioredoxin family and in particular other *Chlamydomonas reinhardtii* TRX sequences remain relatively unexplored (*ie.* TRXx, TRXy, TRXz) but could extend beyond in the thioredoxin superfamily (glutaredoxins, TRX-like, *etc.*). Extending this computational approach to systematically study these sequences could provide invaluable insights into molecular specialization within protein families. Such efforts could pave the way for the development of novel enzymes and proteins with tailored functionalities, addressing challenges in biotechnology and synthetic biology.

Additionally, we used MuLAN as an independent validation tool to investigate the role of the electropositive crown in CrTRXf2. MuLAN confirmed the importance of the six lysines identified by ProfileView and offered a mechanistic perspective by linking these residues to their role in the recognition of FPBase canonical target. This complementary analysis underscores the value of integrating computational methods, such as ProfileView and MuLAN, to derive comprehensive insights into protein structure-function relationships. Looking ahead, the synergy between these methods provides a promising framework for studying not only enzymes but a wide range of proteins.

ProfileView and MuLAN confirmed the previously documented electropositive character that correlates with f-type TRX (Yoshida and Hisabori, 2023) (Yokochi et al., 2019) (Yoshida et al., 2015). We substituted 5 lysines defining TRXf-type onto cytosolic TRXh1, at the positions identified by computational analyses and in agreement with visual comparison of the crystal structures of CrTRXf2 and CrTRXh1 (Lemaire et al., 2018) (Marchand et al., 2019). After purification of resulting TRXχ1, a chimeric TRXh holding the f-type recognition module, we could demonstrate *in vitro* that these mutations enable TRXχ1 to functionally mimick CrTRXf2 in FBPase redox activation. This minimally reconstituted system provides the simplest evidence yet that electropositive surface around the nucleophile cysteine is the actual functional determinant of f-type TRX. Interestingly, TRXχ1 appears more efficient than wild-type CrTRXf2 for CrFBPase activation, suggesting that natural TRX may not operate at biochemical optimum.

By leveraging the power of computational tools and synthetic biology, in association with classical biochemistry we can explore uncharted proteomic landscapes, facilitating the discovery of novel protein variants and engineering protein functional modules for biotechnological applications. Further research might focus on expanding these methods to more complex protein systems, integrating experimental validation, and applying them to other protein families to deepen our understanding of functional determinants and interaction mechanisms.

A practical expansion of our computational/biochemical approach is to achieve the identification of all TRX types on the register of their redox targets, and to test *in vivo* each functional determinant on a generic TRX-folded protein chassis.

## Data Availability

The original contributions presented in the study are included in the article/**Supplementary Material**. Further inquiries can be directed to the corresponding author.

## References

[B1] Abramson J. Adler J. Dunger J. Evans R. Green T. Pritzel A.. (2024). Accurate structure prediction of biomolecular interactions with AlphaFold 3. Nature 630, 493–500. doi: 10.1038/s41586-024-07487-w 38718835 PMC11168924

[B2] Balmer Y. Koller A. Val G. D. Schürmann P. Buchanan B. B. (2004). Proteomics uncovers proteins interacting electrostatically with thioredoxin in chloroplasts. Photosynth Res. 79, 275–280. doi: 10.1023/B:PRES.0000017207.88257.d4 16328793

[B3] Buchanan B. B. (2016). The carbon (formerly dark) reactions of photosynthesis. Photosynth Res. 128, 215–217. doi: 10.1007/s11120-015-0212-z 26704182

[B4] Bunik V. Raddatz G. Lemaire S. Meyer Y. Jacquot J. P. Bisswanger H. (1999). Interaction of thioredoxins with target proteins: role of particular structural elements and electrostatic properties of thioredoxins in their interplay with 2-oxoacid dehydrogenase complexes. Protein Sci. 8, 65–74. doi: 10.1110/ps.8.1.65 10210184 PMC2144114

[B5] Chiadmi M. Navaza A. Miginiac-Maslow M. Jacquot J. P. Cherfils J. (1999). Redox signalling in the chloroplast: structure of oxidized pea fructose-1,6-bisphosphate phosphatase. EMBO J. 18, 6809–6815. doi: 10.1093/emboj/18.23.6809 10581254 PMC1171743

[B6] Decottignies P. Schmitter J. M. Miginiac-Maslow M. Le Maréchal P. Jacquot J. P. Gadal P. (1988). Primary structure of the light-dependent regulatory site of corn NADP-malate dehydrogenase. J. Biol. Chem. 263, 11780–11785. doi: 10.1016/S0021-9258(18)37852-9 3403553

[B7] Droux M. Jacquot J. P. Miginac-Maslow M. Gadal P. Huet J. C. Crawford N. A.. (1987). Ferredoxin-thioredoxin reductase, an iron-sulfur enzyme linking light to enzyme regulation in oxygenic photosynthesis: purification and properties of the enzyme from C3, C4, and cyanobacterial species. Arch. Biochem. Biophys. 252, 426–439. doi: 10.1016/0003-9861(87)90049-X 3028266

[B8] Eklund H. Ingelman M. Söderberg B. O. Uhlin T. Nordlund P. Nikkola M.. (1992). Structure of oxidized bacteriophage T4 glutaredoxin (thioredoxin). Refinement of native and mutant proteins. J. Mol. Biol. 228, 596–618. doi: 10.1016/0022-2836(92)90844-A 1453466

[B9] Elnaggar A. Essam H. Salah-Eldin W. Moustafa W. Elkerdawy M. Rochereau C.. (2023). Ankh: Optimized protein language model unlocks general-purpose modelling. arXiv preprint arXiv:2301.06568. doi: 10.48550/arXiv.2301.06568

[B10] Ingles-Prieto A. Ibarra-Molero B. Delgado-Delgado A. Perez-Jimenez R. Fernandez J. M. Gaucher E. A.. (2013). Conservation of protein structure over four billion years. Structure 21, 1690–1697. doi: 10.1016/j.str.2013.06.020 23932589 PMC3774310

[B11] Jacquot J. P. Gadal P. Nishizawa A. N. Yee B. C. Crawford N. A. Buchanan B. B. (1984). Enzyme regulation in C4 photosynthesis: mechanism of activation of NADP-malate dehydrogenase by reduced thioredoxin. Arch. Biochem. Biophys. 228, 170–178. doi: 10.1016/0003-9861(84)90058-4 6696429

[B12] Ke H. M. Zhang Y. P. Lipscomb W. N. (1990). Crystal structure of fructose-1,6-bisphosphatase complexed with fructose 6-phosphate, AMP, and magnesium. Proc. Natl. Acad. Sci. U.S.A. 87, 5243–5247. doi: 10.1073/pnas.87.14.5243 2164670 PMC54299

[B13] Lancelin J. M. Guilhaudis L. Krimm I. Blackledge M. J. Marion D. Jacquot J. P. (2000). NMR structures of thioredoxin m from the green alga Chlamydomonas reinhardtii. Proteins 41, 334–349. doi: 10.1002/1097-0134(20001115)41:3<334::AID-PROT60>3.0.CO;2-M 11025545

[B14] Laurent T. C. Moore E. C. Reichard P. (1964). Enzymatic synthesis of deoxyribonucleotides. iv. isolation and characterization of thioredoxin, the hydrogen donor from escherichia coli b. J. Biol. Chem. 239, 3436–3444. doi: 10.1016/S0021-9258(18)97742-2 14245400

[B15] Lemaire S. D. Michelet L. Zaffagnini M. Massot V. Issakidis-Bourguet E. (2007). Thioredoxins in chloroplasts. Curr. Genet. 51, 343–365. doi: 10.1007/s00294-007-0128-z 17431629

[B16] Lemaire S. D. Miginiac-Maslow M. (2004). The thioredoxin superfamily in Chlamydomonas reinhardtii. Photosynth Res. 82, 203–220. doi: 10.1007/s11120-004-1091-x 16143836

[B17] Lemaire S. D. Tedesco D. Crozet P. Michelet L. Fermani S. Zaffagnini M.. (2018). Crystal Structure of Chloroplastic Thioredoxin f2 from Chlamydomonas reinhardtii Reveals Distinct Surface Properties. Antioxidants (Basel) 7. doi: 10.3390/antiox7120171 PMC6316601 30477165

[B18] Le Moigne T. Gurrieri L. Crozet P. Marchand C. H. Zaffagnini M. Sparla F.. (2021). Crystal structure of chloroplastic thioredoxin z defines a type-specific target recognition. Plant J. 107, 434–447. doi: 10.1111/tpj.v107.2 33930214

[B19] Lombardi G. Carbone A. (2024). MuLAN: Mutation-driven Light Attention Networks for investigating protein-protein interactions from sequences. bioRxiv: 2024.2008.2024.609515. doi: 10.1101/2024.08.24.609515

[B20] Maeda K. Hägglund P. Finnie C. Svensson B. Henriksen A. (2006). Structural basis for target protein recognition by the protein disulfide reductase thioredoxin. Structure 14, 1701–1710. doi: 10.1016/j.str.2006.09.012 17098195

[B21] Marchand C. H. Fermani S. Rossi J. Gurrieri L. Tedesco D. Henri J.. (2019). Structural and Biochemical Insights into the Reactivity of Thioredoxin h1 from Chlamydomonas reinhardtii. Antioxidants (Basel) 8. doi: 10.3390/antiox8010010 PMC6356897 30609656

[B22] Michelet L. Zaffagnini M. Morisse S. Sparla F. Pérez-Pérez M. E. Francia F.. (2013). Redox regulation of the Calvin-Benson cycle: something old, something new. Front. Plant Sci. 4, 470. doi: 10.3389/fpls.2013.00470 24324475 PMC3838966

[B23] Perez-Jimenez R. Inglés-Prieto A. Zhao Z. M. Sanchez-Romero I. Alegre-Cebollada J. Kosuri P.. (2011). Single-molecule paleoenzymology probes the chemistry of resurrected enzymes. Nat. Struct. Mol. Biol. 18, 592–596. doi: 10.1038/nsmb.2020 21460845 PMC3087858

[B24] Qin J. Clore G. M. Gronenborn A. M. (1994). The high-resolution three-dimensional solution structures of the oxidized and reduced states of human thioredoxin. Structure 2, 503–522. doi: 10.1016/S0969-2126(00)00051-4 7922028

[B25] Schürmann P. Buchanan B. B. (1975). Role of ferredoxin in the activation of sedoheptulose diphosphatase in isolated chloroplasts. Biochim. Biophys. Acta 376, 189–192. doi: 10.1016/0005-2728(75)90217-0 235981

[B26] Schürmann P. Wolosiuk R. A. (1978). Studies on the regulatory properties of chloroplast fructose-1,6-bisphosphatase. Biochim. Biophys. Acta 522, 130–138. doi: 10.1016/0005-2744(78)90329-7 202319

[B27] Sekiguchi T. Yoshida K. Okegawa Y. Motohashi K. Wakabayashi K. I. Hisabori T. (2020). Chloroplast ATP synthase is reduced by both f-type and m-type thioredoxins. Biochim. Biophys. Acta Bioenerg 1861, 148261. doi: 10.1016/j.bbabio.2020.148261 32659266

[B28] Steinegger M. Söding J. (2017). MMseqs2 enables sensitive protein sequence searching for the analysis of massive data sets. Nat. Biotechnol. 35, 1026–1028. doi: 10.1038/nbt.3988 29035372

[B29] Vicedomini R. Bouly J. P. Laine E. Falciatore A. Carbone A. (2022). Multiple profile models extract features from protein sequence data and resolve functional diversity of very different protein families. Mol. Biol. Evol. 39. doi: 10.1093/molbev/msac070 PMC9016551 35353898

[B30] Villeret V. Huang S. Zhang Y. Xue Y. Lipscomb W. N. (1995). Crystal structure of spinach chloroplast fructose-1, 6-bisphosphatase at 2.8. ANG. resolution. Biochemistry 34, 4299–4306. doi: 10.1021/bi00013a019 7703243

[B31] Yokochi Y. Sugiura K. Takemura K. Yoshida K. Hara S. Wakabayashi K. I.. (2019). Impact of key residues within chloroplast thioredoxin-f on recognition for reduction and oxidation of target proteins. J. Biol. Chem. 294, 17437–17450. doi: 10.1074/jbc.RA119.010401 31597700 PMC6873186

[B32] Yoshida K. Hara S. Hisabori T. (2015). Thioredoxin selectivity for thiol-based redox regulation of target proteins in chloroplasts. J. Biol. Chem. 290, 14278–14288. doi: 10.1074/jbc.M115.647545 25878252 PMC4505498

[B33] Yoshida K. Hisabori T. (2023). Current insights into the redox regulation network in plant chloroplasts. Plant Cell Physiol. 64, 704–715. doi: 10.1093/pcp/pcad049 37225393 PMC10351500

[B34] Zaffagnini M. Fermani S. Marchand C. H. Costa A. Sparla F. Rouhier N.. (2019). Redox homeostasis in photosynthetic organisms: novel and established thiol-based molecular mechanisms. Antioxid Redox Signal 31, 155–210. doi: 10.1089/ars.2018.7617 30499304

