# Supplementary figures and images for: Functional recoding of Chlamydomonas reinhardtii thioredoxin type-h into photosynthetic type-f by switching selectivity determinants
Source: Front Plant Sci. 2025 Mar 6;16:1554272. doi: 10.3389/fpls.2025.1554272 (PMC11922929; doi:10.3389/fpls.2025.1554272)

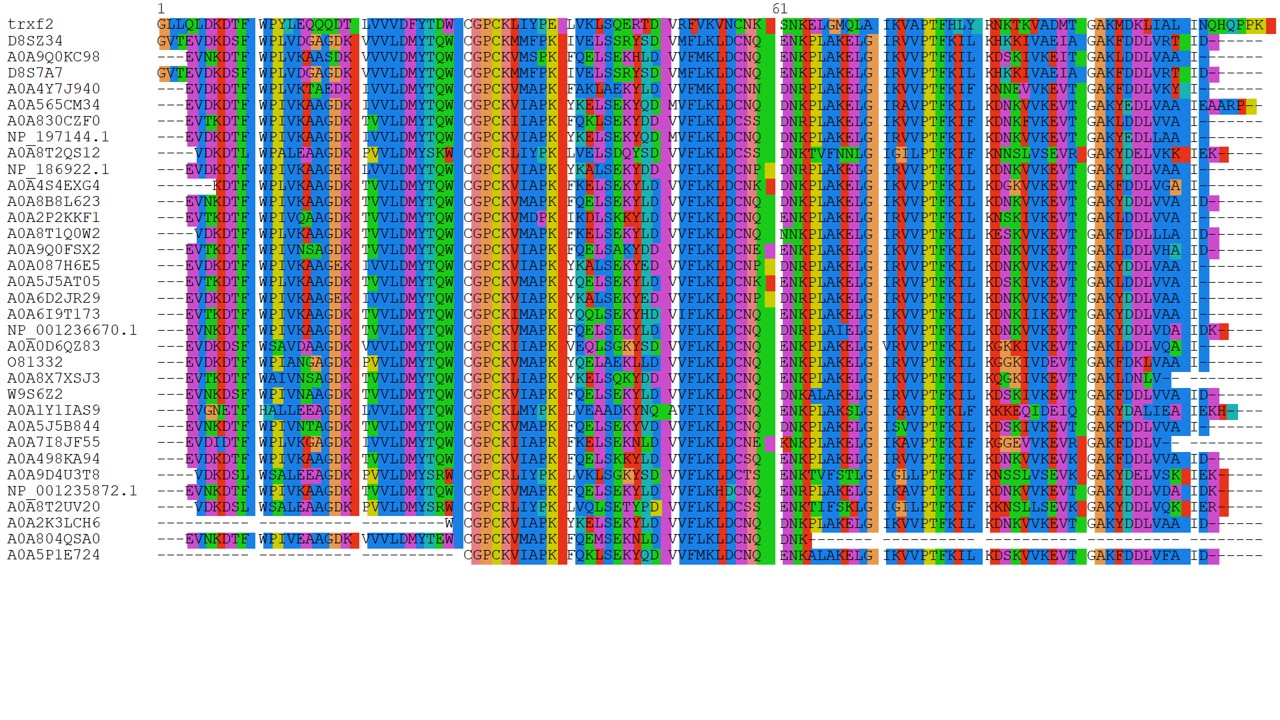

Supplement: Supplementary file 1 [file Image1.jpg]
